# Supplementary material for: Ecological networks are more sensitive to plant than to animal extinction under climate change
Source: Nat Commun. 2016 Dec 23;7:13965. doi: 10.1038/ncomms13965 (PMC5196430; doi:10.1038/ncomms13965)
Supplement: Supplementary Information — Supplementary Figures and Supplementary Tables [file ncomms13965-s1.pdf]

Supplementary Table 1 | Biotic specialization, climatic niche breadth and vulnerability to climate change. Results from linear-mixed effects models of the effects of (**a, b**) realized climatic niche breadth (climatic hypervolume[60], OMI climatic niche breadth[61]) and (**c, d**) projected changes in climatic suitability (RCP 6.0, RCP 8.5 scenarios[65], year 2070) on the effective number of interaction partners[66] and complementary specialization d'[67] of plants and animals (n = 295 plant and 414 animal species). Given are estimates of main and interaction effects (β) with their standard error (SE) across 13 mutualistic networks from central Europe; main effects of climatic predictors reflect the estimated effect for animals and '*plant*' indicates the contrast between plant and animal species. In the interest of comparability, continuous predictor variables were z-transformed prior to the analysis. Models account for random variation due to network identity, plant and animal taxonomy on model intercepts. Models of climatic suitability change were weighted by the accuracy (TSSmax value[64]) of the respective species distribution model; p-values of estimates were derived by Kenward-Roger approximation (***, $p < 0.001$; **, $p < 0.01$; *, $p < 0.05$).

	Effective number of partners [log]		d'	
	β	SE	β	SE
**(a) Climatic hypervolume [square-root]**				
Intercept	0.645 ***	0.068	0.313 ***	0.018
Hypervolume	0.142 ***	0.031	-0.004	0.007
Plant	0.431 ***	0.080	0.019	0.012
Hypervolume x Plant	-0.163 **	0.048	0.005	0.011
**(b) OMI climatic niche breadth**				
Intercept	0.653 ***	0.068	0.313 ***	0.018
Niche breadth	0.145 ***	0.033	-0.002	0.007
Plant	0.420 ***	0.081	0.019	0.012
Niche breadth x Plant	-0.140 **	0.049	0.009	0.011
**(c) Climatic suitability change [RCP 6.0]**				
Intercept	0.680 ***	0.069	0.313 ***	0.018
Suitability change [RCP 6.0]	0.104 **	0.033	-0.006	0.007
Plant	0.396 ***	0.083	0.023	0.013
Suitability change [RCP 6.0] x Plant	-0.116 *	0.052	-0.002	0.011
**(d) Climatic suitability change [RCP 8.5]**				
Intercept	0.681 ***	0.069	0.313 ***	0.018
Suitability change [RCP 8.5]	0.107 **	0.035	-0.005	0.008
Plant	0.392 ***	0.083	0.023	0.013
Suitability change [RCP 8.5] x Plant	-0.114 *	0.051	-0.004	0.011

Supplementary Table 2 | Biotic specialization, climatic niche breadth and vulnerability to climate change for 13 mutualistic networks. Given are estimates for the statistical association between (**a, b**) realized climatic niche breadth (climatic hypervolume[60], OMI climatic niche breadth[61]) and (**c, d**) projected changes in climatic suitability (RCP 6.0, RCP 8.5 scenarios[65], year 2070) and two measures of biotic specialization for plants and animals. Specialization equals the number of effective partners[66] or complementary specialization *d′*[67]. Network-specific estimates (β) were derived from random intercept and slope models of the respective predictor variable on network identity, run separately for plants and animals. Models also account for effects of plant and animal taxonomy on model intercepts. Models of climatic suitability change were weighted by the accuracy (TSSmax value[64]) of the respective species distribution model. In the interest of comparability, climatic predictors were *z*-transformed prior to analysis. Values are highlighted if the overall slope across networks was significantly larger than 0.

Network identity	**(a)** Climatic hypervolume				**(b)** OMI climatic niche breadth			
	Effective number of partners [log]		*d′*		Effective number of partners [log]		*d′*	
	Plant	Animal	Plant	Animal	Plant	Animal	Plant	Animal
	β	β	β	β	β	β	β	β
P1	-0.052	**0.143**	0.003	-0.008	-0.024	**0.150**	0.008	-0.005
P2	0.037	**0.157**	-0.013	-0.004	0.056	**0.200**	-0.008	-0.007
P3	0.030	**0.131**	0.003	-0.009	0.049	**0.145**	0.008	-0.004
P4	-0.020	**0.116**	0.007	-0.004	0.006	**0.095**	0.011	-0.007
P5	-0.001	**0.136**	-0.003	-0.006	0.016	**0.160**	0.002	-0.006
P6	-0.062	**0.112**	0.012	-0.008	-0.031	**0.134**	0.016	-0.005
P7	-0.043	**0.118**	-0.003	-0.008	-0.018	**0.050**	0.002	-0.005
P8	-0.041	**0.128**	0.008	-0.009	-0.016	**0.109**	0.013	-0.004
S1	-0.012	**0.122**	-0.022	0.001	0.014	**0.098**	-0.016	-0.010
S2	0.006	**0.139**	-0.011	-0.002	0.029	**0.161**	-0.005	-0.009
S3	-0.007	**0.163**	-0.021	-0.007	0.010	**0.215**	-0.016	-0.006
S4	-0.036	**0.101**	0.011	-0.009	-0.007	**0.030**	0.016	-0.004
S5	-0.031	**0.136**	-0.006	-0.007	-0.003	**0.128**	-0.001	-0.005

Network identity	**(c)** Climatic suitability change (RCP 6.0)				**(d)** Climatic suitability change (RCP 8.5)			
	Effective number of partners [log]		*d′*		Effective number of partners [log]		*d′*	
	Plant	Animal	Plant	Animal	Plant	Animal	Plant	Animal
	β	*β*	*β*	*β*	*β*	*β*	*β*	*β*
P1	-0.011	**0.026**	-0.013	-0.011	-0.005	**0.031**	-0.014	-0.012
P2	-0.023	**0.008**	0.002	-0.004	-0.032	**0.003**	0.000	-0.004
P3	-0.013	**0.087**	-0.014	-0.015	-0.006	**0.083**	-0.014	-0.014
P4	-0.010	**0.147**	-0.016	-0.003	-0.003	**0.157**	-0.017	-0.003
P5	-0.012	**0.072**	-0.009	-0.009	-0.008	**0.073**	-0.010	-0.007
P6	-0.008	**0.090**	-0.022	-0.013	0.001	**0.099**	-0.022	-0.011
P7	-0.009	**0.208**	-0.009	-0.017	-0.003	**0.191**	-0.010	-0.013
P8	-0.012	**0.110**	-0.018	-0.015	-0.011	**0.106**	-0.019	-0.014
S1	-0.013	**0.130**	0.011	0.008	-0.011	**0.134**	0.009	0.010
S2	-0.013	**0.207**	0.001	0.001	-0.008	**0.198**	-0.001	0.003
S3	-0.013	**0.058**	0.010	-0.012	-0.009	**0.055**	0.008	-0.010
S4	-0.010	**0.076**	-0.023	-0.013	-0.004	**0.072**	-0.023	-0.014
S5	-0.011	**0.062**	-0.004	-0.013	-0.006	**0.059**	-0.005	-0.011

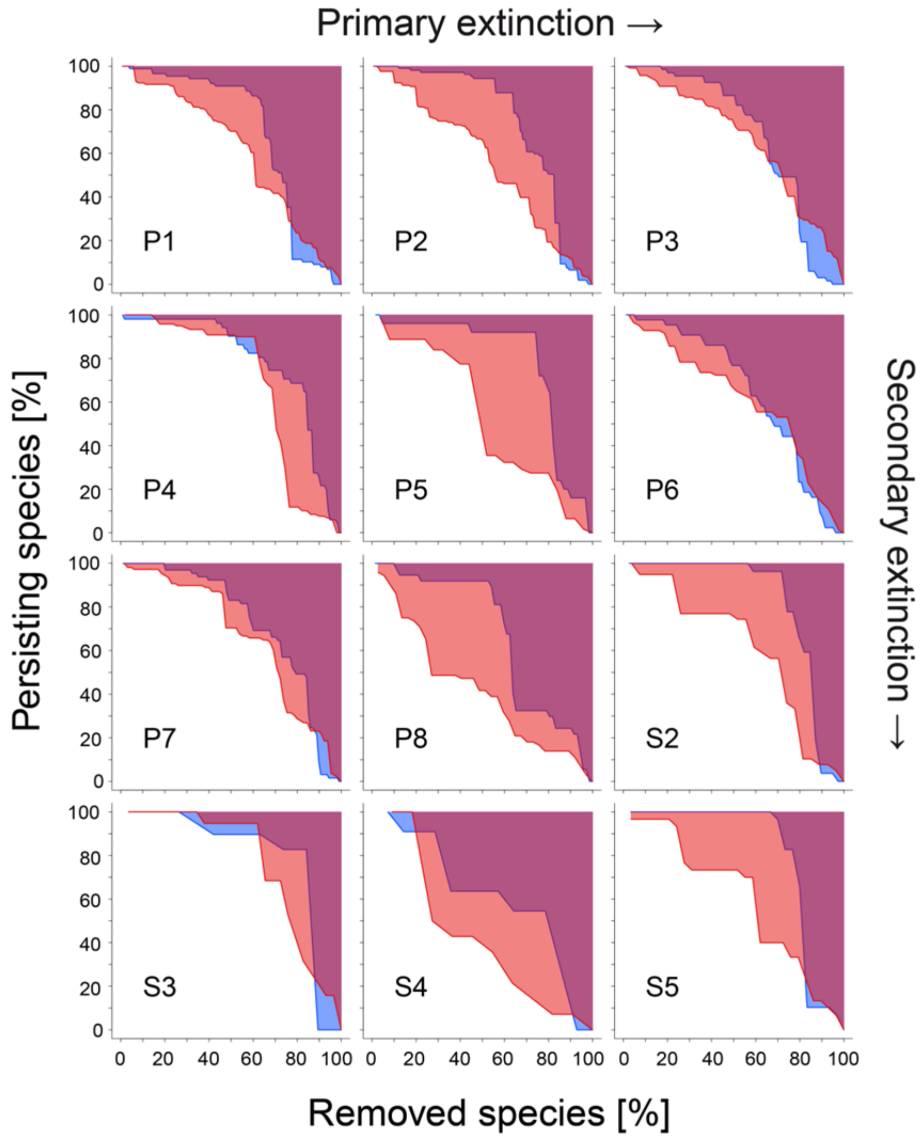

Supplementary Figure 1 | Secondary animal and plant extinction under climate change. Secondary extinction plots show network sensitivity to species extinction (filled area above the extinction curve) for eight pollination (P1-P8) and four seed-dispersal networks (S2-S5): secondary animal extinction in response to plant extinction (red) and secondary plant extinction in response to animal extinction (blue); overlapping areas of secondary animal and secondary plant extinction are shown in purple. In general, sensitivity to plant extinction (i.e. the red area) was larger than sensitivity to animal extinction (i.e. the blue area). Here we assume an extinction threshold of 50% interaction loss before secondary extinction is triggered and a species' flexibility to reallocate 50% of lost interactions to persisting partners (constrained rewiring). Note that secondary extinction plots for seed-dispersal network S1 are shown in Fig. 2.

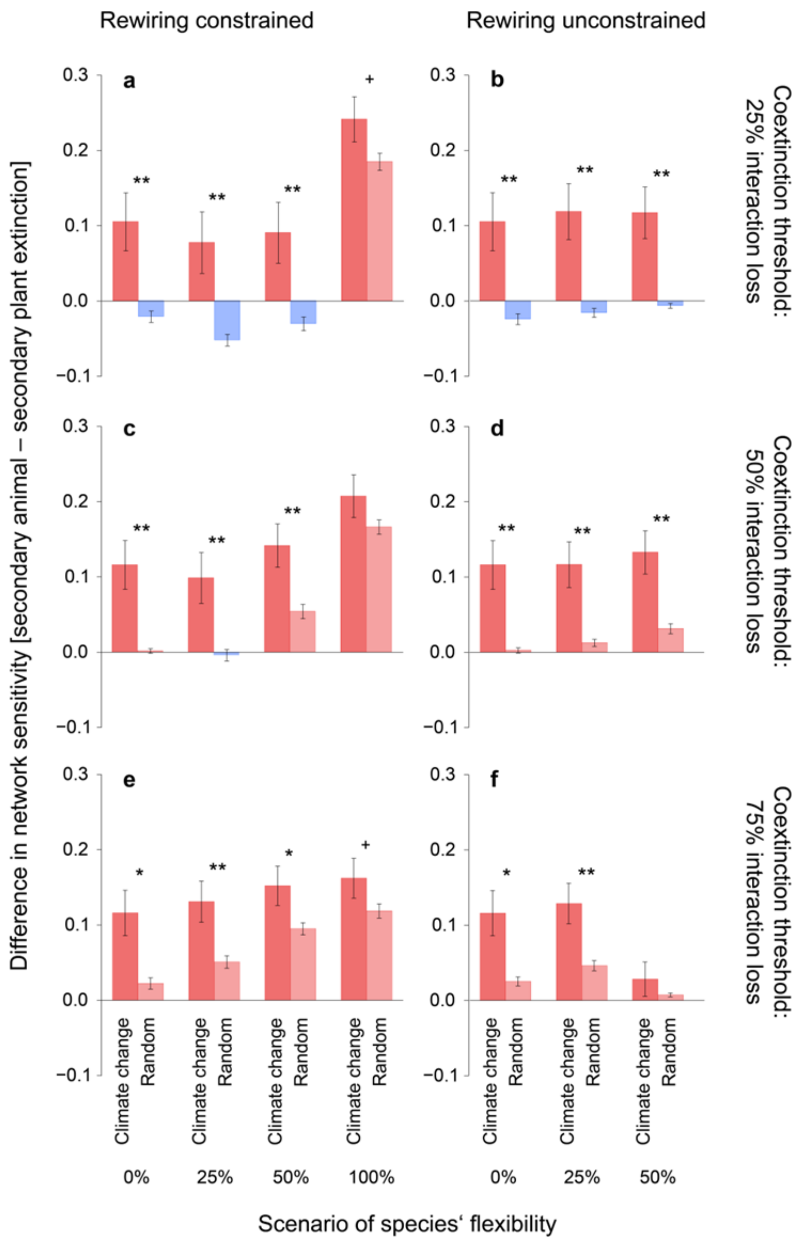

Supplementary Figure 2 | Differences in sensitivity to species extinction across 13 mutualistic networks. Shown are differences in network sensitivity to plant versus animal extinction for different scenarios of species' sensitivity to coextinction, rewiring capacity and flexibility. Coextinction thresholds varied between (**a, b**) 25%, (**c, d**) 50% and (**e, f**) 75% of interaction loss. Species were able to rewire interactions (**a, c, e**) to persisting partners (constrained rewiring) or (**b, d, f**) to all persisting species in each network (unconstrained rewiring). Flexibility values (0%, 25%, 50%, 100%) indicate the proportion of lost interactions that was reallocated to other species in the respective scenario. Shown are mean differences (±1 SE) across the 13 pollination and seed-dispersal networks between the impact of plant versus animal extinction; values larger than 0 (red bars) indicate a higher risk of secondary animal than secondary plant extinction and values smaller than 0 (blue bars) indicate the opposite. Secondary animal versus secondary plant extinction was compared between climate change and random extinction using two-sided, pair-wise t-tests [**, $p < 0.01$; *, $p < 0.05$; $^{+}$, $p < 0.1$]. Climatic projections of the models of species' vulnerability to climate change follow the RCP 6.0 scenario (compare Figure 3 for the RCP 8.5 scenario).

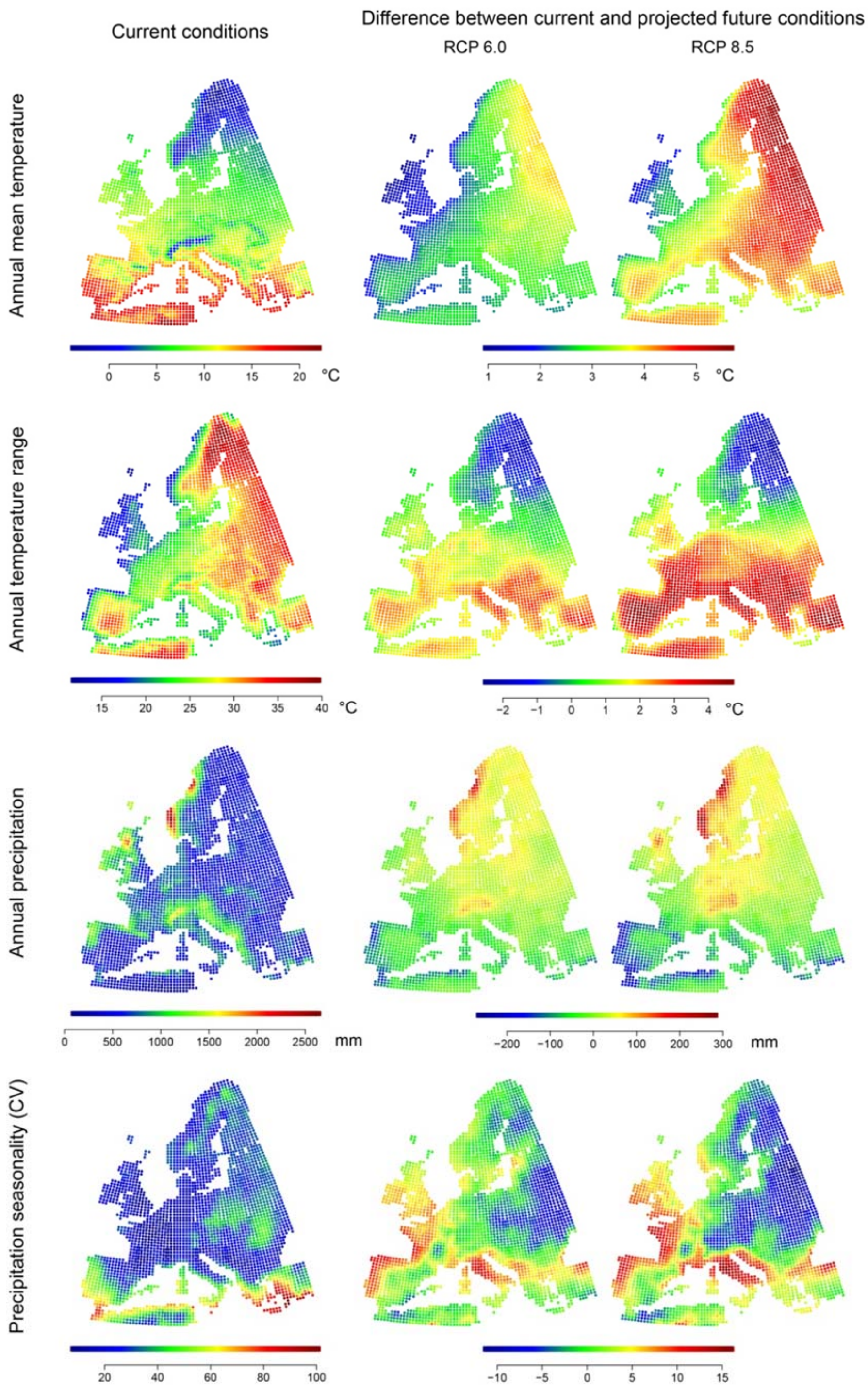

Supplementary Figure 3 | Current and projected future climatic conditions. Shown are current conditions (average for 1950-2000) and the difference between current and projected future conditions for annual mean temperature, temperature annual range, annual precipitation and precipitation seasonality across the European CGRS grid (3024 equal-area cells with a spatial resolution of 50 km x 50 km); future conditions are projected according to two Representative Concentration Pathways (RCP 6.0, RCP 8.5) for the year 2070 (averaged for 2061-2080), averaged across two general circulation models (CCSM4, MIROC5[59,65]).

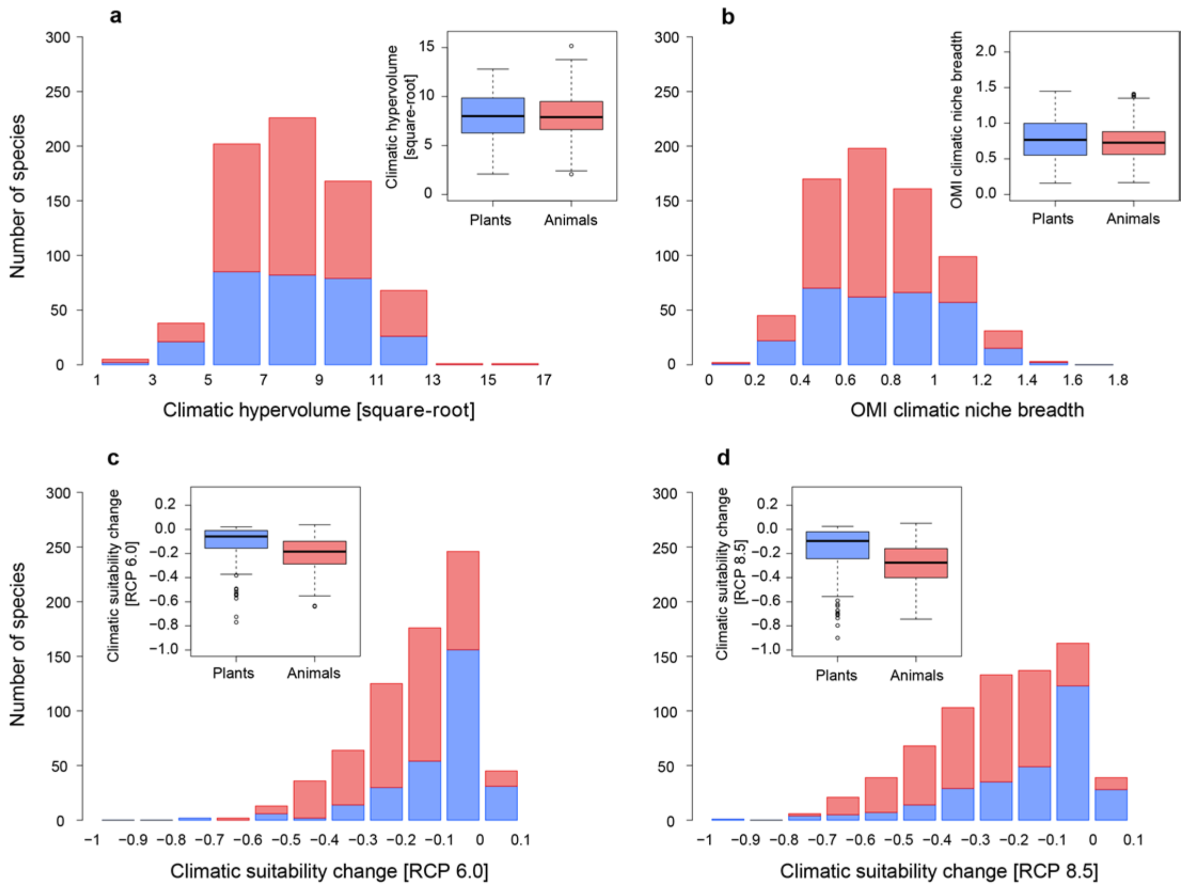

Supplementary Figure 4 | Climatic niche breadth and vulnerability to climate change for plant and animal species. Frequency distributions of (**a, b**) realized climatic niche breadth (climatic hypervolume[60], OMI climatic niche breadth[61]), and (**c, d**) projected future changes in climatic suitability (RCP 6.0, RCP 8.5 scenarios[65], year 2070) for plant (blue) and animal (red) species (295 plant, 196 bee, 70 butterfly, 97 hoverfly, 51 bird species). Inserts show box-plots comparing the distribution of the respective variable between plants and animals; lines across boxes are medians, boxes indicate 25th and 75th percentiles, whiskers indicate the data range, and circles are outliers.
